# Responder analyses and the assessment of a clinically relevant treatment effect

**DOI:** 10.1186/1745-6215-8-31

**Published:** 2007-10-25

**Authors:** Steven M Snapinn, Qi Jiang

**Affiliations:** 1 Steven Snapinn and Qi Jiang, Amgen Inc., One Amgen Center Drive, 24-2-C, Thousand Oaks, CA 91320, USA

## Abstract

Ideally, a clinical trial should be able to demonstrate not only a statistically significant improvement in the primary efficacy endpoint, but also that the magnitude of the effect is clinically relevant. One proposed approach to address this question is a responder analysis, in which a continuous primary efficacy measure is dichotomized into "responders" and "non-responders." In this paper we discuss various weaknesses with this approach, including a potentially large cost in statistical efficiency, as well as its failure to achieve its main goal. We propose an approach in which the assessments of statistical significance and clinical relevance are separated.

## Background

A clinical endpoint used to determine the efficacy of an experimental treatment can be measured on a variety of scales, including a continuous scale, an ordinal scale, or a binary scale. One important goal common to many clinical trials is determining whether or not the effect of the experimental treatment is significantly better than that of the control treatment, and this goal can be achieved using measurements based on any of these scales. However, it is not only important to assess statistical significance, but also to assess the clinical relevance of the effect, and the assessment of clinical relevance has received much less attention in the statistical literature. The purpose of this paper is to discuss some of the issues associated with the assessment of clinical relevance, focusing in particular on an approach known as the "responder analysis" that involves dichotomizing a continuous or ordinal variable into a binary variable.

One potential approach to assess clinical relevance is to define a clinically relevant effect, and test the null hypothesis that the true effect is of this size or less versus the hypothesis that the true effect is greater than the clinically relevant effect. For example, suppose that the clinical endpoint is a continuous variable, X, such that larger values represent better efficacy, and that there is interest in the mean difference in this endpoint, *μ*, between the experimental treatment and the control. Note that X could represent a measurement taken at the conclusion of the trial or a change in that measurement from its baseline value. The typical null hypothesis (assuming one-sided testing) is that of no difference, or *μ *≤ 0, versus the alternative hypothesis *μ *> 0. However, if one were to define a minimum clinically important difference, *μ*_0_, then one could test the null hypothesis *μ *≤ *μ*_0 _versus the alternative hypothesis *μ *> *μ*_0_. This hypothesis is sometimes referred to as "super superiority," and a statistically significant result in this case would imply both statistical significance and clinical relevance. However, the "super superiority" null hypothesis is not frequently used in practice, probably due to concerns with decreased power.

Probably the most common approach to assess clinically relevance is to compare the observed treatment effect with the minimum clinically important difference. That is, statistical significance is based on a test of the null hypothesis of no difference, and clinical relevance is concluded if the observed treatment effect, μ^
 MathType@MTEF@5@5@+=feaafiart1ev1aaatCvAUfKttLearuWrP9MDH5MBPbIqV92AaeXatLxBI9gBaebbnrfifHhDYfgasaacH8akY=wiFfYdH8Gipec8Eeeu0xXdbba9frFj0=OqFfea0dXdd9vqai=hGuQ8kuc9pgc9s8qqaq=dirpe0xb9q8qiLsFr0=vr0=vr0dc8meaabaqaciaacaGaaeqabaqabeGadaaakeaaiiGacuWF8oqBgaqcaaaa@2E79@, is greater than *μ*_0_.

Another approach also in common use, which we refer to as a responder analysis, is based on defining a threshold value above which a subject is considered to be a "responder," and below which a subject is considered to be a "non-responder." If we let x_0 _represent the threshold value, then

Y={1if X≥x00if X<x0
 MathType@MTEF@5@5@+=feaafiart1ev1aaatCvAUfKttLearuWrP9MDH5MBPbIqV92AaeXatLxBI9gBaebbnrfifHhDYfgasaacH8akY=wiFfYdH8Gipec8Eeeu0xXdbba9frFj0=OqFfea0dXdd9vqai=hGuQ8kuc9pgc9s8qqaq=dirpe0xb9q8qiLsFr0=vr0=vr0dc8meaabaqaciaacaGaaeqabaqabeGadaaakeaacqWGzbqwcqGH9aqpdaGabeqaauaabaqaciaaaeaacqaIXaqmaeaacqqGPbqAcqqGMbGzcqqGGaaicqWGybawcqGHLjYScqWG4baEdaWgaaWcbaGaeGimaadabeaaaOqaaiabicdaWaqaaiabbMgaPjabbAgaMjabbccaGiabdIfayjabgYda8iabdIha4naaBaaaleaacqaIWaamaeqaaaaaaOGaay5Eaaaaaa@4351@

is a binary variable indicating whether or not the subject is a responder. Now let p_X _and p_C _be the response rates in the experimental group and the control group, respectively. The null hypothesis for the responder analysis, therefore, is p_X _≤ p_C_, and the alternative is p_X _> p_C_. As in the case of "super superiority," if the responder null hypothesis is rejected then both statistical significance and clinical relevance are concluded.

Some examples of this approach in the medical literature include the following. Eron et al [1] compared fosamprenavir-ritonavir to lopinavir-ritoavir in HIV-1-infected subjects, and one of the primary endpoints was the achievement of HIV-1 RNA less than 400 copies per mL at week 48. Tyring et al [2] compared etanercept with placebo in subjects with moderate to severe psoriasis, and the primary endpoint was a 75% or greater improvement from baseline in psoriasis area and severity index score (PASI 75) at week 12. Edwards et al [3] compared oral methotrexate, rituximab, rituximab plus cyclophosphamide and rituximab plus methotrexate in subjects with active rheumatoid arthritis, and the primary endpoint was the achievement of an ACR 50 response at week 24. An ACR 50 response was defined as an improvement of at least 50 percent from baseline in counts of both tender and swollen joints, as well as in three of five other disease-activity measures. McMillan-Price et al [4] compared 4 reduced-fat, high-fiber diets in overweight or obese young adults, and the reported results included a comparison of the proportions of subjects who lost 5% or more of body weight over 12 weeks.

This responder approach is also described in regulatory guidance documents. For example, a recent draft guidance from the FDA on patient-reported outcomes [5] specifically endorsed the responder analysis as an alternative approach to assessing clinical relevance:

Page 19, Defining a minimum important difference:*"Many PRO instruments are able to detect mean changes that are very small; accordingly it is important to consider whether such changes are meaningful. Therefore, it is appropriate for a critical distinction to be made between the mean effect seen (and what effect might be considered important) and a change in an individual that would be considered important, perhaps leading to a definition of a responder."*

Page 20, Definition of responders: *"There may be situations where it is more reasonable to characterize the meaningfulness of an individual's response to treatment than a group's response, and there may be interest in characterizing an individual patient as a responder to treatment, based upon prespecified criteria backed by empirically derived evidence supporting the responder definition as a measure of benefit. Such examples include categorizing a patient as a responder based upon a prespecified change from baseline on one or more scales; a change in score of a certain size or greater (e.g., a 2-point change on an 8-point scale); or a percent change from baseline."*

Page 25, Planning for Study Interpretation: *"In some cases, the FDA may request an a priori definition of the minimum observed difference between treatment group means (i.e., MID) that will serve as a benchmark to interpret whether study findings are conclusive. In other cases, the FDA may request an a priori definition of a treatment responder that can be applied to individual patient changes over time."*

In addition, a recent guidance from the Committee for Medicinal Products for Human Use [6] concerning non-inferiority trials included the following:

Page 6, Demonstrating Efficacy: *"Establishing a clinically relevant benefit over placebo is accomplished by considering the point estimate of the difference between the test product and placebo and assessing its clinical relevance, either using the original scale or by considering responder rates.... A judgement must be made regarding whether the difference seen is clinically useful."*

Although the responder analysis is in common use, it has substantial disadvantages, and even its benefits do not stand up to careful scrutiny. Therefore, the purpose of this paper is to examine this approach in some detail.

## Methods

### Cost in Power

One well-known disadvantage of the responder analysis is reduced power relative to an analysis on the original scale [7-9]. Consider a simple model in which the measurement, X, is a normally distributed variable with known variance. Without loss of generality, assume unit variance, a true mean value in the control group of -*μ*/2, and a true mean value in the experimental group of *μ*/2. Under this model, power and sample size for a comparison of the means of the two groups are completely determined by *μ*, but the power and sample size for a comparison of responder rates depend both on *μ *and the threshold value, x_0_.

Table 1 illustrates the impact on the required sample size for 90% power under this model. Clearly, the major factor affecting sample size for both approaches was *μ*, the true mean difference between the two groups. As *μ *increased from 0.2 to 1.0, the sample size required to detect a mean difference between groups decreased from 526 subjects per group to 21. The threshold value, x_0_, also had a major impact on the sample size for the responder analysis. The required sample size was minimized when the threshold value fell midway between the mean values for the two treatment groups; *i.e*., x_0 _= 0. However, even in this case, the sample size requirement for the responder analysis was far greater than for the test of a mean difference, with a relative increase of approximately 60%. As the threshold value moved away from the midway point, the difference between the sample size requirements for the two approaches increased greatly. For example, with a mean difference between groups of 0.2 units and a threshold value of x_0 _= 2, which corresponds to responder rates of p_X _= 2.9% and p_C _= 1.8%, the sample size requirement for the responder analysis is 4053 subjects per group, or nearly an 8-fold increase over the 526 subjects per group required to detect a difference in means.

**Table 1:** Sample Size Required for 90% Power to Detect a Difference in Means or a Difference in Responder Rates

*μ*	x_0_	Responder Rates		Per Group Sample	Per Group Sample
		p_X_	p_C_	Size – Difference in Means	Size – Difference in Rates
0.2	0	54.0%	46.0%	526	827
0.2	1	18.4%	13.6%	526	1204
0.2	2	2.9%	1.8%	526	4053

0.5	0	59.9%	40.1%	84	133
0.5	1	22.7%	10.6%	84	197
0.5	2	4.0%	1.2%	84	689

1.0	0	69.1%	30.9%	21	34
1.0	1	30.9%	6.7%	21	53
1.0	2	6.7%	0.6%	21	200

Note that under different models the impact on power and sample size could be smaller, and indeed there may be cases where the responder analysis has greater power than an analysis based on the continuous variable. However, these situations usually lead to difficulties in interpretation of the results whichever analysis is used, as we'll discuss below.

While this impact on sample size in the normally distributed case is quite large, it could certainly be acceptable if the benefit of the responder analysis, that is, its ability to help ensure a clinically important treatment effect, were real. However, as we'll discuss below, this purported benefit is largely illusory.

### Other Problems with the Responder Analysis

Perhaps the main problem with the responder analysis is the arbitrary nature of the definition of a response [10]. Clearly, there are some situations where the achievement of a certain value on a continuous scale has enormous clinical implications, such as when a test value is used as the basis for a decision on hospital admission or surgical intervention. In these cases, an analysis of the response rates would be highly relevant despite any cost in power. Alternatively, the clinical event itself (i.e., the hospitalization or the surgery) would make an appropriate endpoint for determining the treatment effect. However, in most cases the cutoff value used to determine a responder is an arbitrary point on a continuous scale. For example, in the cases presented earlier, there is probably very little clinical difference between the achievement of 399 versus 401 copies per mL of HIV-1 RNA, or between a weight loss of 4.9% versus 5.1%. However, there may be great differences in clinical relevance *within *the responder and non-responder groups; for example, the differences between a weight loss of 5.1% versus 20%, and between a loss of 4.9% versus a gain of 10%, are probably quite relevant. In fact, it's the loss of information associated with lumping these groups together that leads to the decreased efficiency and increased sample size requirements described above.

It is also interesting to consider the inconsistent application of the responder analysis. There are some disease areas where the use of a responder analysis is expected (for example, the use of a specific ACR cutoff such as ACR 50 in rheumatoid arthritis) and others where it is unusual (for example, percent change from baseline in bone mineral density in subjects with osteoporosis). Given the substantial cost in efficiency associated with the responder analysis it seems that there should be a clear rationale for the situations where it is or is not appropriate. The simple desire to assess the clinical relevance of the treatment effect must not be a sufficient criterion, since clinical relevance is important in every clinical setting, while the use of the responder analysis is sporadic. However, there does not appear to be any other clear criterion.

It is also interesting to consider the meaning of the term "responder" in the context of a controlled clinical trial. In many clinical settings the outcome measurement may improve over time for reasons unrelated to the study treatment. Some causes might include regression to the mean, measurement error, natural history of the disease, or therapies taken by the subject other than the study therapy. Of course, this is the primary reason for the inclusion of a control group. However, the term "responder" seems to imply a belief that the improvement in the outcome measurement on a particular subject was caused by the experimental treatment. A more appropriate definition of response (i.e., a causal effect) would be based on the outcome of an individual subject when taking the experimental treatment relative to the outcome that subject would have experienced with a placebo. However, this type of information is not available in standard parallel-group clinical trials. In fact, in such trials the true causal effect of the treatment (i.e., the effect beyond that which a placebo would have produced) can only be assessed by a comparison of the groups.

Consider the case of a placebo-controlled trial in which a large fraction of the subjects in the experimental group responded (say, 30%), but the response rate was identical in the placebo group. In such a case it would seem likely that none of the "responses" were due to the activity of the experimental therapy, and so it would seem inappropriate to refer to any of these subjects as "responders."

Finally, recall one of the key drivers for the use of a responder analysis from the FDA guidance document: trials may have the ability to detect small mean changes that are not clinically relevant. However, the responder analysis actually suffers from exactly the same problem. Note that the hypotheses *μ *≤ 0 and p_X _≤ p_C _are both null hypotheses of no difference between groups; therefore, in both cases, rejecting the null hypothesis simply rules out a zero difference between groups. In fact, in the case of distributions in the treatment and control populations that differ only in location (such as in the case of two normally distributed distributions with identical variance), the two hypotheses are identical: rejecting either one implies that the other is rejected as well. Just as in a test for mean differences between treatment groups, with a large enough sample size any arbitrarily small difference between treatment groups in response rates can result in statistical significance, regardless of the rigor or lack of rigor in the definition of a responder. Therefore, rejection of the null hypothesis of equal response rates in no way guarantees that the effect of the treatment is clinically meaningful.

## Results and discussion

The goal of determining whether or not a treatment effect is clinically meaningful is certainly an important one, but the responder analysis alone does not accomplish it. Just as in the test for a mean difference between groups, the null hypothesis for the responder analysis is of no difference between groups. Therefore, rejection of either null hypothesis simply allows one to conclude that there is a non-zero difference between the groups, not that the difference is clinically meaningful. Given the substantial cost in efficiency associated with this analysis in many cases, the responder analysis should typically be avoided as the primary analysis approach.

How, then, does one achieve the goal of determining whether or not a treatment effect is clinically meaningful? Unless one is willing to define a "super superiority" hypothesis, then the approach should be to separate the questions of statistical significance and clinical importance. In order to achieve this goal, the first step is to determine whether or not the treatment effect is real through the use of a statistical hypothesis test. This should typically be based on the original, continuous scale, but if there is reason to believe that the responder analysis has greater power than the analysis of the continuous data then the responder analysis could be considered. The next step is to determine clinical importance by examination of the mean difference between groups, as well as by examination of response rates, possibly using various response definitions.

**Figure 1:**
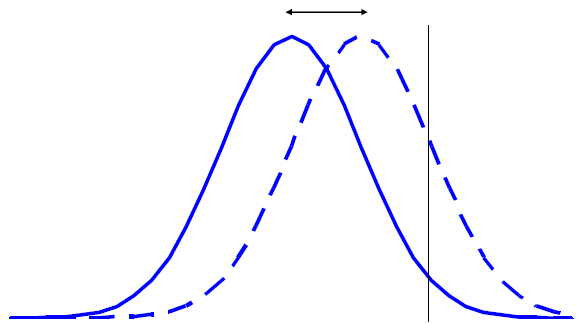
Distribution of Outcomes in the Experimental Group (Dashed Line) Has Greater Mean Value Than Control Group (Solid Line) And Greater Proportion of Responders.

**Figure 2:**
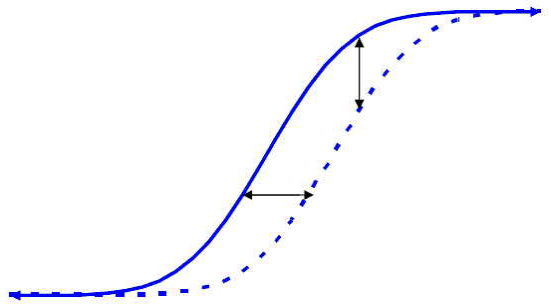
Cumulative Distribution Functions for Two Treatment Groups When the Outcome Variable Distributions Differ in Mean But Not Variance; Horizontal Displacement Represents the Mean Difference and Vertical Displacement Represents the Difference in Response Rates.

However, the assessment of both statistical significance and clinical importance can be complicated by the nature of the distributions of response. First, take the simplest case of two normal distributions with identical variances but different means. Figure 1 illustrates the density functions for the experimental treatment (the dashed line) and the control (the solid line), and Figure 2 illustrates their cumulative distribution functions. (In both figures the x-axis is the continuous response variable, with greater values indicating greater efficacy, and the vertical line in Figure 1 represents a hypothetical threshold.) In this case, the two null hypotheses (*μ *≤ 0 and p_X _≤ p_C_) are identical, and the existence of a mean difference implies the existence of difference in response rates, and vice versa; therefore, the same test, such as a t-test, could be used to test either hypothesis. The consistent horizontal separation between the distribution functions (Figure 2) suggests that the benefit on the continuous scale was consistent among subjects, although it should be recognized that other explanations are possible. If the assumption of a consistent benefit among subjects seems reasonable, the mean difference between groups would be an appropriate summary of the treatment benefit, and its magnitude should be used to help determine clinical relevance. In addition, the vertical distance between the curves is a measure of the difference in responder rates for a specific threshold value. Since the vertical difference will always differ for different threshold values (unless the curves completely overlap and the difference is always zero), it makes sense to evaluate multiple threshold values to help assess clinical relevance.

**Figure 3:**
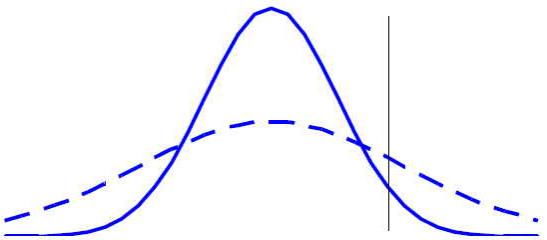
Distribution of Outcomes in the Experimental Group (Dashed Line) Has Equal Mean Value to That of the Control Group (Solid Line), But a Greater Proportion of Responders.

**Figure 4:**
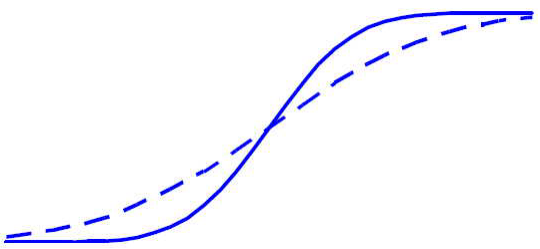
Cumulative Distribution Functions for Two Treatment Groups When the Outcome Variable Distributions Have the Same Mean But Different Variance.

Contrast this with Figures 3 and 4, in which the variances differ between the two groups, resulting in an inconsistency between the mean difference and the difference in response rates. In this case, the means of the two distributions are identical, while the experimental treatment has a higher response rate. This is obviously a case where an analysis based on response rates would have greater power than an analysis based on the mean difference. However, the analysis and interpretation in this case are considerably more complicated than in the case of equal variances. First, the assessment of statistical significance based on the continuous variable must take into account the nature of the distributions. (Note that the common t-test assumes equal variances, and so would be inappropriate in this case.) In addition, the observed mean difference between groups does not appear to be a satisfactory summary statistic in this case since examination of Figure 4 suggests that the treatment effect is inconsistent from subject to subject. In fact, it appears as though some subjects benefit from the treatment while others are harmed. Assessment of clinical significance in this case can be difficult. While examination of response rates can be helpful, is should be emphasized that, due to the arbitrary nature of any specific cutoff value, various definitions of response should be used.

Of course, the distributions of responses may differ in considerably more complex ways than illustrated here. In addition, due to limited sample sizes, it may be difficult to tell whether the horizontal difference between two empirical distribution functions suggests a consistent effect from patient to patient. Despite these limitations, examination of the empirical distribution functions should provide valuable information that simple comparisons of means or response rates do not provide.

Until now we have assumed the original response variable is continuous, but the same arguments apply when the response variable is ordinal. A responder analysis can be defined by dichotomizing the ordinal scale, but an analysis of the ordinal variable using, say, a proportional odds model, will typically have considerably greater power.

## Conclusion

In conclusion, the main disadvantage of the responder analysis, its reduced efficiency relative to an analysis of the original continuous variable, is well-known, but the other weaknesses of the responder analysis highlighted in the paper are less well-known. The recommended approach to assess statistical significance and clinical relevance should be sequential, with statistical significance based on the most powerful approach (usually using the continuous variable) and clinical relevance based on examination of the mean difference between groups and on response rates. It is also important to examine the cumulative distribution functions to help assess the consistency of response among subjects.

## Competing interests

The authors are employees of and hold stock in Amgen, a biotechnology company that conducts clinical trials, some of which involve dichotomizing a continuous variable.
